# A Measurement Setup and Automated Calculation Method to Determine the Charge Injection Capacity of Implantable Microelectrodes

**DOI:** 10.3390/s18124152

**Published:** 2018-11-27

**Authors:** Ana Cisnal, Juan-Carlos Fraile, Javier Pérez-Turiel, Victor Muñoz-Martinez, Carsten Müller, Frank R. Ihmig

**Affiliations:** 1 Fraunhofer-Institut für Biomedizinische Technik (IBMT), Department of Biomedical Microsystems, 66280 Sulzbach/Saar, Germany; ana.cisnal@hotmail.com (A.C.); carsten.mueller@ibmt.fraunhofer.de (C.M.); frank.ihmig@ibmt.fraunhofer.de (F.R.I.); 2 ITAP, Universidad de Valladolid, Paseo del Cauce 59, 47011 Valladolid, Spain; jcfraile@eii.uva.es; 3 Escuela de Ingenierías Industriales, Universidad de Málaga, Doctor Ortiz Ramos s/n, 29071 Málaga, Spain; vfmm@uma.es

**Keywords:** access voltage, cyclic voltammetry, voltage transient measurements, charge injection capacity, charge storage capacity, surface roughness

## Abstract

The design of safe stimulation protocols for functional electrostimulation requires knowledge of the “maximum reversible charge injection capacity” of the implantable microelectrodes. One of the main difficulties encountered in characterizing such microelectrodes is the calculation of the access voltage *V_a_*. This paper proposes a method to calculate *V_a_* that does not require prior knowledge of the overpotential terms and of the electrolyte (or excitable tissue) resistance, which is an advantage for in vivo electrochemical characterization of microelectrodes. To validate this method, we compare the calculated results with those obtained from conventional methods for characterizing three flexible platinum microelectrodes by cyclic voltammetry and voltage transient measurements. This paper presents the experimental setup, the required instrumentation, and the signal processing.

## 1. Introduction

Functional electrostimulation is a technique which consists in applying an electric pulse sequence to a muscle or nerve, to generate body movements or to restore voluntary motor functions which are paralyzed due to injury to the central nervous system. Applications of functional electrostimulation include the control of limb prostheses [1], treating spinal cord injuries [2,3,4], retina and cochlear prostheses for sensory problems involving vision [5,6] and hearing [7,8,9], and deep brain stimulation for neural problems, including Parkinson’s disease [10], involuntary movements, and psychiatric diseases such as depression and obsessive compulsive disorder [11,12].

For functional restoration, electrical pulses are applied via electrodes that are implanted on a muscle or nerve [13]. Some of the emerging biomedical applications require the development of smaller electrodes because they must provide high spatial resolution; i.e., the application of stimuli must be highly selective. However, such microelectrodes are required to deliver charge densities that exceed the traditional damage threshold, and consequently the charge density is close to safe limits at clinically effective levels [14]. Microelectrodes are characterized by high electrical impedance and not enough charge injection capacity for some therapeutic applications because of their small geometric surface area (GSA). For this reason, alloys or coatings with high surface roughness are commonly used to significantly increase the effective surface area (ESA) of the electrode and, therefore, the amount of injected charge [15,16].

The most common method of charge injection in the context of functional electrostimulation is known as the galvanostatic or current-controlled method [17], which is based on the use of two-phase balanced charge electric pulses that inject the same magnitude of anodic and cathodic charge, resulting in zero charge transfer in a stimulation pulse [18]. For physiological reasons, the first phase of stimulation is typically cathodic and is used to obtain the desired function. The second phase is anodic and is used to reverse the electrochemical processes that occurred in the first phase [19,20].

The magnitude of the current applied to the electrode must allow safe and effective stimulation [21]. Effective stimulation implies that the current intensity suffices to inject the necessary charge load into the target tissue to cause the desired physiological effect in the patient. However, to avoid damage to the tissue or electrode, the current intensity must not be excessive, so that products formed in the first phase of the electrical pulse can return to their original composition upon reversing the current direction [17].

The design of safe stimulation protocols requires knowledge of the maximum charge that an electrode can inject to ensure that all reactions that occur in the electrolyte are reversible. This parameter is known as the “maximum reversible charge injection capacity” (CIC) and is determined by studying the electrochemical behavior of the electrode.

The potential region in which all reactions occurring in the electrolyte are reversible (i.e., in which no anodic or cathodic oxidation or reduction occurs in water) is commonly referred to as the “electrochemical potential window”. This parameter is determined by cyclic voltammetry (CV), which is a three-electrode measurement in which the potential of the working electrode (WE), with respect to the reference electrode (RE), is swept cyclically at a constant predefined rate between two potential limits while allowing current to flow between the WE and the counter electrode (CE) [22]. This technique can identify the type of reactions that occur in the electrolyte and their degree of reversibility, because the current is proportional to the type of reaction [23]. It is also possible to determine the cathodic charge storage capacity (CSC_c_), which is defined as the total amount of reversible charge available in the cathodic phase of the stimulation pulse. It is calculated by integrating the cathodic current over a potential range whose limits correspond to the electrochemical potential window [24].

The maximum reversible charge injection capacity per unit area is determined by analyzing voltage transients (VTs). This technique consists of applying a controlled current pulse to the WE while measuring its voltage relative to the RE. It is used to determine the maximum polarization at the electrode/electrolyte interface, which can be compared with the potential limits (potentials that are considered safe for polarizing the WE), which are typically the limits of the electrochemical potential window [15].

The difficulty with the VT technique is the precise calculation of the access voltage *V_a_* required to determine the maximum safe electrode polarization. The access voltage is associated with the voltages that do not influence the polarization of the electrode; namely, the concentration overpotential and the voltage drop due to electrolyte resistance [15]. The concentration overpotential can be calculated through the Nernst equation [25], whereas the voltage drop due to the electrolyte resistance is calculated based on the electrical model of the electrode/electrolyte interface [26,27,28]. However, for in vivo characterization, it has to be noted that the electrical properties of excitable tissue cells are isotropic and inhomogeneous [29].

The voltage drop in the electrolyte is much greater than the voltage drop caused by concentration-related factors. For this reason, and taking into account the difficulty of calculating these two factors, it is common practice to calculate the access voltage by considering only the voltage drop in the electrolyte and disregarding the voltage drop due to concentration-related factors.

**Figure 1:**
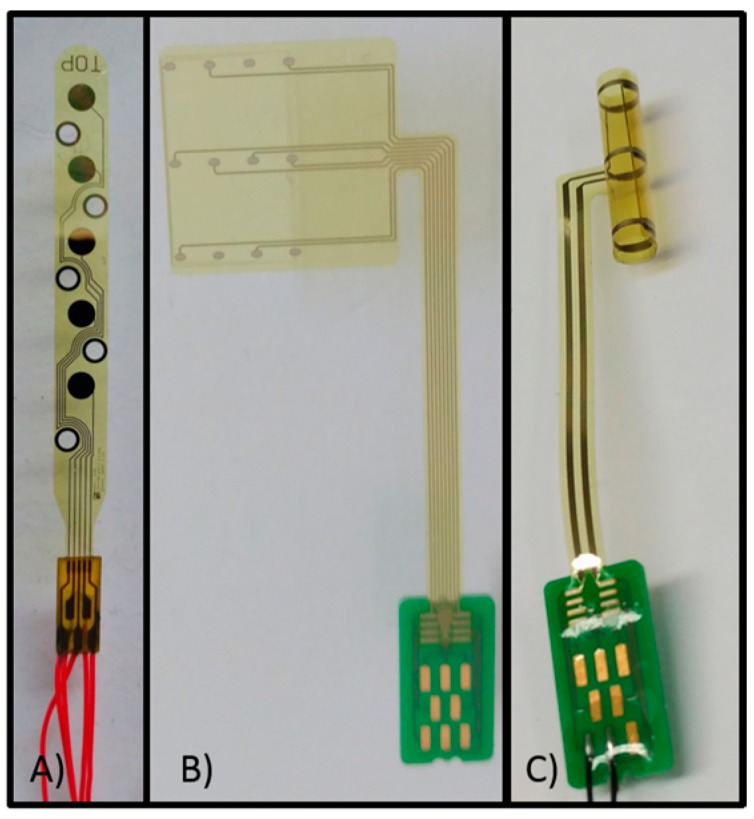
Three flexible Pt microelectrodes with circular contacts, designed and fabricated at Fraunhofer IBMT: (**A**) planar Pt electrode with 5 contacts, (**B**) cuff Pt electrode with 12 contacts, (**C**) cuff microporous Pt electrode with 12 contacts.

To address this problem, we propose a method to calculate the access, in which the access voltage approaches the voltage drop in the electrolyte, because it does not require prior knowledge of the overpotential terms and of the electrolyte (or excitable tissue) resistance. This approach is advantageous for in vivo electrochemical characterization of microelectrodes. In addition, applying the proposed method does not require introducing the inter-pulse period for the calculation of the access voltage, as is proposed in some conventional methods. To validate the proposed method, we compare the results with those obtained by using conventional methods to characterize three flexible platinum microelectrodes (see Figure 1).

We present herein the experimental setup, the required instrumentation, and the processing of the measured potential transients for determining the access voltage and the CIC.

## 2. Materials and Methods

The CIC and potential limits were calculated for three different platinum electrodes using VTs and CV, respectively. We developed a new method for calculating the access voltage to improve the determination of the CIC. This method was compared to one conventional method.

A. Electrodes

In this work, we characterized flexible Pt microelectrodes listed in Table 1 and shown in Figure 1. These are suitable for neural stimulation and recording.

The planar electrode (electrode A; Figure 1a) is formed from five 0.5 cm diameter Pt contacts based on a 20 µm thick polyimide film and with an inter-contact distance of 4 mm. Besides neural stimulation, this electrode design can be used for the acquisition of muscle activity, which is an exemplary application for its recording capability. In this case, the electrode design allows for epimysial implantation (the electrode is placed underneath the epimysium, which is a sheath of fibrous elastic tissue surrounding a muscle) [30].

The cuff Pt electrode (electrode B, Figure 1b), which is not in the final cuff shape, and the cuff microporous Pt electrode (electrode C, Figure 1c) are highly flexible and made of polyimide with 12 integrated Pt contacts having an inter-contact distance of 5 mm. Cuff electrodes are implanted around the nerve, making selective neuromuscular activation possible. The entire structure is designed with physical properties and dimensions that avoid compression and stretch [31].

B. Electrochemistry setup

CV and VT measurements were performed using a Pt auxiliary electrode and a room temperature saline isotonic solution as electrolyte. CV measurements were made by using commercially available interconnected modules from Solartron Analytical (Leicester, UK): 1260A Impedance/Gain-Phase Analyzer and 1287A Potentiostat/Galvanostat. The impedance analyzer measures frequency response over a wide range (from 10 to 32 MHz) and with high resolution (0.015 ppm). Five cycles were executed to ensure that the electrode reached its stable state. These five cycles were recorded at a sweep rate of 0.1 V/s, beginning at the open circuit potential and sweeping first in the positive direction.

**Figure 2:**
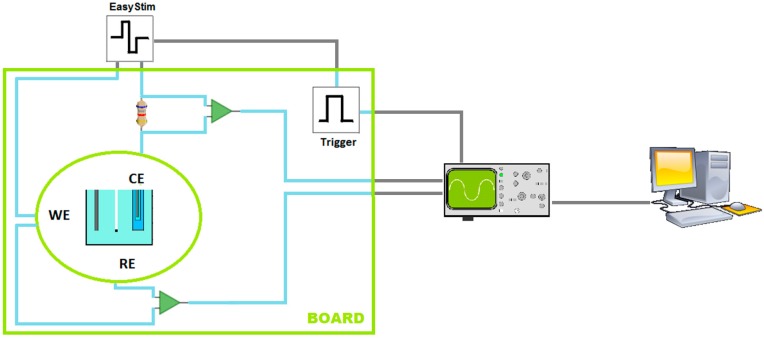
VT measurements—Simplified diagram of measurement setup: WE is the working electrode, RE is the reference electrode, CE is the counter electrode. Custom circuit board and oscilloscope to capture signals. Stimulator (EasyStim) and computer for signal processing.

VT measurements were done by applying a current-controlled stimulation pulse to a WE while its potential with respect to the RE is recorded. Figure 2 shows a simplified diagram of the setup developed to make VT measurements for characterizing electrodes.

*Custom circuit board and pulse stimulator*

**Figure 3:**
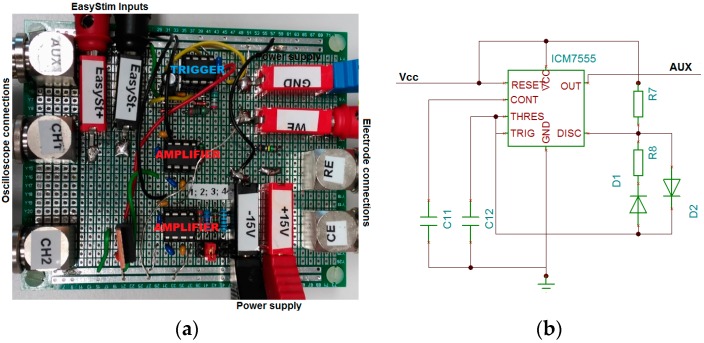
(**a**) Custom circuit board for VT measurements. (**b**) Configuration of timer ICM7555 IPA (Intersil, Milpitas, CA, USA).

Figure 3a shows the custom circuit board developed at Fraunhofer IBMT to perform VT measurements.

The function of the external trigger (AUX) is to simultaneously trigger both, the oscilloscope and the biphasic current pulse stimulator (EasyStim). This stimulator excites the WE while it is externally triggered at a frequency of 50 Hz with a pulse width of 200 µs and a current amplitude that can be varied from 0.05 to 2 mA.

The general-purpose and inexpensive timer ICM7555 IPA (Intersil, Milpitas, CA, USA) is used to implement the external trigger. The linear voltage regulator LM340-5 (National Semiconductor, Santa Clara, CA, USA) provides a stable +5 V DC signal to power the timer. Two high-speed diodes, particularly the 1N41748, are also necessary for the implementation. The trigger signal has a free-running oscillation frequency of 50 Hz, the rise-time of the pulse is 50 µs, and the duty cycle is 0.25%. Figure 3b shows the configuration implemented for the timer to run a work cycle of less than 50%.

The potential drop across the resistance in series with the WE and CE and the WE potential with respect to the RE are the two differential signals to be amplified. High speed is required for amplifying the transient potential to achieve a good-quality waveform (CH1). For this reason, we use the instrument amplifier INA111AP (Texas Instruments, Dallas, TX, USA), which is characterized by a slew rate of 17 V/µs.

The current through the electrodes is measured indirectly from the voltage drop across the 15.4 Ω resistor in series with the electrodes (CH2). The measurement of the current amplitude does not require special characteristics, so we use the general-purpose instrument amplifier INA121 (Texas Instruments).

*Oscilloscope*

We used a TDS5052 Digital Oscilloscope (Tektronix, Beaverton, OR, USA) which has two input channels and an auxiliary third channel. It offers the possibility to transmit data to a computer. We used the “average acquisition mode”, which removes uncorrelated noise from the input signal. The oscilloscope acquires *N* consecutive waveforms. The final waveform displayed is the averaged result of the previous acquisitions. The averaged result is the average value for each recorded point over *N* acquisitions. In our case, *N* = 8. Although this mode requires a repeating signal, it reduces the random noise without compromising bandwidth. The auxiliary signal of the oscilloscope is configured to be the trigger signal AUX for both channels CH1 and CH2. The resolution of the oscilloscope is set to 5000 points.

*Computer*

The connection between oscilloscope and computer is accomplished by the TEKVISA interface, which is the implementation of the Virtual Instrument Software Architecture (VISA) Application Programming Interface by Tektronix. VISA is an input-output library designed by VXIplug&play System Alliance that provides a common standard for the software connection between different provider systems on the same platform.

Locally, TEKVISA allows controlling the instruments through a general purpose interface bus, USB and serial (RS-232) interfaces, or remotely via an Ethernet local area network connection. In this setup, the connection is made by using the local area network, which uses a VXI-11 protocol.

The physical support used to establish the local area communication consists of two Ethernet wires; one connected to the PC and one to the oscilloscope. A RJ45 modular serial adapter or switch is used to connect the PC to the oscilloscope.

With the setup shown in Figure 2, a stimulation signal is applied to the WE. The VTs on the WE with respect to the RE are recorded by the oscilloscope and imported into the computer. The signals processing was done in OriginPro 2015 (OriginLab Corp., Northampton, MA, USA) using the Origin C programming language.

The signal $V_{We-Re}\left(t\right)$ (the WE potential with respect to the RE) is filtered to smooth the signal and remove random noise by using a Savitzky-Golay filter (five points, second order). The signal $I_{We-Ce}\left(t\right)$ (the current from the WE to the CE, which is the stimulation current in mA) is filtered by a Savitzky-Golay filter (30 points, second order) to calculate the amplitude of the stimulation current.

C. Measurements of voltage transients

**Figure 4:**
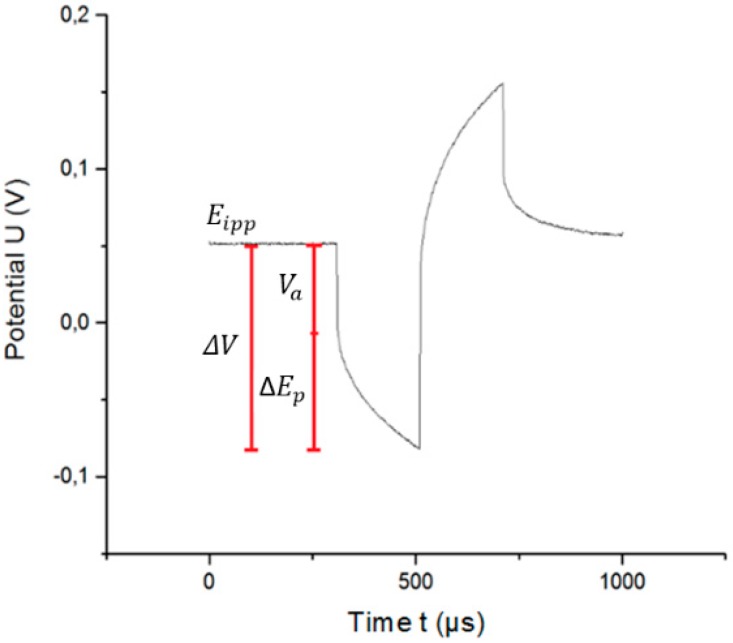
VT curve of electrode excited by a symmetric, biphasic current pulse. Pulse width is 200 µs and frequency is 50 Hz.

Figure 4 shows the VT curve of a WE to which a symmetric, biphasic current is applied with respect to the RE.

Figure 4 shows several elements that contribute to the overall voltage drop ΔV:

(i) $E_{ipp}$: Potential at the WE at the pulse onset.
(ii) $\Delta E_{p}$: Polarization across the electrode/electrolyte interface. It is defined as the sum of the activation overpotential $\eta_{a}$, and the potential due to the electrode being taken out of its equilibrium position $\Delta E_{o}$:
  (1) $$\Delta E_{p}=\eta_{a}+\Delta E_{o}$$
(iii) $V_{a}$: Access voltage, defined as the instantaneous change in potential at the beginning of a pulse or immediately after the pulse. It is calculated as the sum of the voltages that do not influence the electrode polarization (i.e., the concentration overpotential $\eta_{c}$ and voltage drop across the electrolyte resistance $i_{c}R_{c}$). Specifically:
  (2) $$V_{a}=i_{c}R_{c}+\eta_{c}$$
(iv) ΔV: Voltage transient, which depends on the voltage drop $i_{c}R_{c}$ due to the electrolyte resistance, the concentration overpotential $\eta_{c}$, the activation overpotential $\eta_{a}$, and the potential due to the electrode being taken out of its equilibrium position $\Delta E_{o}$:
  (3) $$\Delta V=i_{c}R_{c}+\eta_{c}+\eta_{a}+\Delta E_{o}$$
  The maximum polarization $E_{m}$ is defined by [15]:
  (4) $$E_{m}=E_{ipp}+\Delta E_{p}=E_{ipp}+\left(\Delta V-V_{a}\right)$$
  which depends on the electrode, electrolyte, and stimulation signal, with $E_{ma}$ being the most positive (anodic) and $E_{mc}$ being the most negative (cathodic) polarization. $\Delta E_{p}$ is obtained by subtracting $V_{a}$ from the VT-measured ΔV. One of the main problems for determining $E_{ma}$ and $E_{mc}$ is the difficulty of accurately measuring $V_{a}$.

### 2.1. Conventional and Proposed Methods to Calculate the Access Voltage

Guaranteeing a safe reversible charge injection is essential to achieve a useful functional electrical stimulation. The knowledge of the electrode polarization, and hence of the access voltage is critical to operate within a safe range of injected charge. Moreover, accurate measuring the maximum charge injection capacity acquires greater relevance when the therapeutic stimulation involves the use of small dimension electrodes due to the need of provide large injected charges which approach the safety limits.

The access voltage depends on three factors: concentration overpotential in the electrolyte, amplitude of applied excitation current, and electrolyte resistance. The access voltage is difficult to calculate accurately due to the complexity of determining the concentration overpotential in the electrolyte. Difficulties are also encountered in calculating electrolyte (or excitable tissue) resistance in in vivo experiments where tissue resistivity is heterogeneous [29] and it changes after electrode implantation [32]. Furthermore, identifying the access voltage precisely was found to be a hard issue due to factors such as current-pulse rise times and stray capacitance [15,22,33].

The literature proposes two conventional methods for calculating $V_{a}$. The first method approximates $V_{a}$ as the voltage drop across the electrolyte resistance because the contribution of the overpotential terms is very small compared with the voltage drop across the electrolyte resistance [34,35,36]. The second method introduces a small interpulse period between the cathodic and anodic phase of the biphasic and rectangular current pulse to facilitate the identification of the access voltage [33,37,38,39]. The charge-injection limits depend on the anodic bias level and current density delivered during pulsing which may be inconsistent with the interpulse period introduced by this method [39].

In this paper, we propose a method for automated calculation of the access voltage associated with the voltage drop in the electrolyte and the overpotential terms. This method is expected to be advantageous compared with the first conventional method described because it does not neglect the overpotential terms and does not require a priori knowledge of the electrolyte resistance. Furthermore, the proposed method does not require introducing the interpulse period for calculating the access voltage, as is the case for the second conventional technique.

The following describes the first conventional method that approximates $V_{a}$ as the voltage drop in the electrolyte resistance (Method 1) and describes the proposed method for calculating the access voltage (Method 2). We use Method 1 to validate the results because both methods are valid when the electrodes are excited by a biphasic current pulse, both balanced and without interpulse period. Next, we use these two methods to calculate $V_{a}$ to characterize the three microelectrodes listed in Table 1 and to validate the results obtained with the proposed method.

(i) Method 1: Conventional method

This conventional method approximates the access voltage as the voltage drop in the electrolyte resistance. The access voltage is defined as the sum of the concentration overpotential and the voltage drop across the electrolyte resistance. However, the contribution of the overpotential terms is very small compared with the Ohmic drop in the electrolyte. For this reason, the overpotential terms are commonly neglected, so the voltage drop approximates the access voltage across the electrolyte resistance ($V_{Rs}$):

(5) $$V_{a}\approx V_{Rs}=iR_{s}$$

The solution resistance $R_{s}$ for circular, non-coated microelectrodes is [40]:

(6) $$R_{s}=\frac{\rho }{\pi r}$$

However, to determine the solution resistance of a microelectrode whose roughness factor is not zero, it is necessary to calculate the equivalent electrical model of an electrode/electrolyte interface [24,25,26].

This method does not allow the determination of the maximum reversible charge injection capacity accurately since it neglects the concentration overpotential that was found to correspond to 20% of the access voltage on AIROF electrodes [41]. Another disadvantage is the need to know the heterogenous tissue resistance which undergoes changes once the electrode has been implanted. Besides, Ir Oxide and PEDOT electrodes suffer changes in the ohmic resistance during the stimulation [15]. The method proposed below overcomes these drawbacks: it allows to obtain a more precise access voltage and it does not present problems associated to tissue resistance, a critical issue for materials whose impedance is voltage-dependent or in vivo applications.

(ii) Method 2: Proposed method

The proposed method calculates the access voltage based on the definition by Cogan [15]: “… the access voltage $V_{a}$ is taken as the near-instantaneous voltage change at either the onset of the current pulse or immediately after the current pulse is terminated.” The access voltage may be quantified by deriving the WE’s potential transient, because the derivation measures the rate of the potential change. For discrete data, the central difference formula should be used to approximate the derivation if the step h is constant and sufficiently small:

(7) $$f^{\prime }\left(x_{i}\right)=\frac{f\left(x_{i+1}\right)-f\left(x_{i-1}\right)}{2h}$$

**Figure 5:**
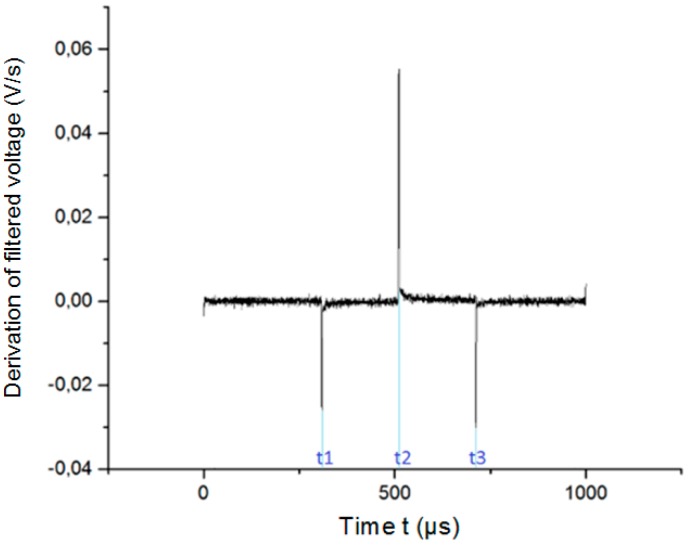
Time derivation of Pt electrode potential transients for 200 µs stimulation signal.

The access voltage can be calculated as the sum of two consecutive points of the transient potential:

(8) $$V_{a}=\sum \left[f\left(x_{i+1}\right)-f(x_{i})\right]=\sum 2h\left[f^{\prime }\left(x_{i+1}\right)+f^{\prime }(x_{i})\right]+f\left(x_{i-1}\right)-f\left(x_{i+2}\right),$$

when the requirements stated in Equations (9) and (10) are met. In Equation (8) $f\left(x_{i}\right)$ is the potential of the WE with respect to the RE at each moment of time $x_{i},f^{\prime }\left(x_{i}\right)$ is the time derivation of $f\left(x_{i}\right)$, and *h* is the step. The access voltage depends on the time of change and the limit of the derivation of the transient. The times at which the stimulation signal changes sign (*t*_1_, *t*_2_, and *t*_3_) are calculated from the time derivation of the potential transient. Figure 5 shows the time derivation of the potential transients of a Pt electrode excited by a cathodic-first, charge-balanced biphasic symmetric current pulse with a 200 μs pulse width and a frequency of 50 Hz. The times *t*_1_, *t*_2_, and *t*_3_ correspond to the times where the three peaks appear in the derivation. The pulse width *p*_w_ is calculated as *p*_w_ = (*t*_3_ − *t*_1_)/2.

The minimum derivation must be calculated when there are no potential changes or when the changes are only due to signal noise. The potential transient can be unstable in the first and last instants of the samples. For this reason, the derivation may be unstable, too. To solve this problem when performing computational calculations, the analysis of the time interval known as false time (ft), with a duration of the first and last 15 µs of the waveform, is avoided. The minimum value of the derivation is called the *lower limit* and it is calculated during the first interpulse period; in particular, from $x_{i}=ft$ to $x_{i}=t_{1}-ft$.

**Figure 6:**
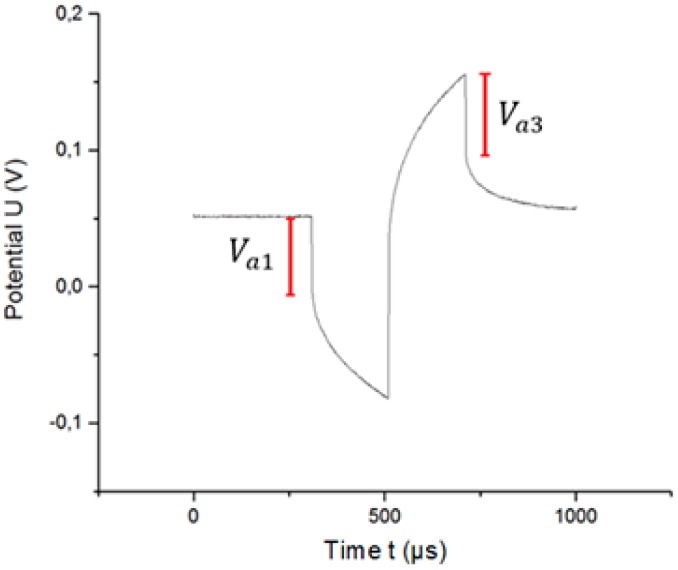
Different access voltages (*V*_*a*1_ and *V*_*a*3_) in the potential transient.

Conversely, the access voltage is defined as the near-instantaneous change in potential. For this reason, we define a time called the “time of change” $t_{c}$ = 5 µs. This means that the potential change due to the access voltage may occur, at most, over a time interval of 10 µs. Figure 6 shows the calculated access voltages.

(9) $$V_{a1}=\mathrm{abs}\sum \left[f\left(x_{i+1}\right)-f(x_{i})\right]\;\mathrm{if}\;f^{\prime }\left(x_{i}\right)<\mathrm{lower}\;\mathrm{limit}\;\mathrm{and}\;t_{1}-t_{c}<x_{i}<t_{1}+t_{c}$$

(10) $$V_{a3}=\mathrm{abs}\sum \left[f\left(x_{i+1}\right)-f(x_{i})\right]\;\mathrm{if}\;f^{\prime }\left(x_{i}\right)<\mathrm{lower}\;\mathrm{limit}\;\mathrm{and}\;t_{3}-t_{c}<x_{i}<t_{3}+t_{c}$$

The access voltage is considered to be a positive value, so we must calculate the absolute value (abs) for the access voltages $V_{a1}$ and $V_{a3}$, because the derivation is negative and consequently the difference is also negative.

### 2.2. Graphical Correction of the Access Voltage

**Figure 7:**
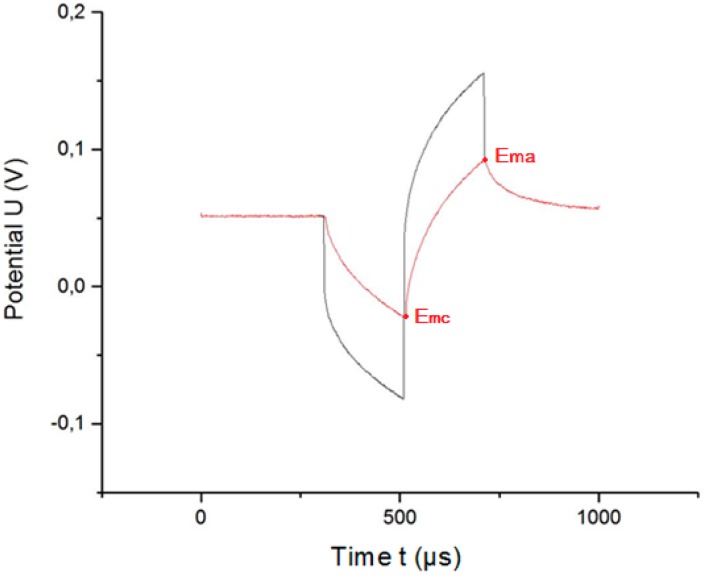
Graphical correction of the access voltage. Black line is original potential transient, and red line is corrected potential transient.

The calculation of the maximum reversible charge injection by an electrode requires a priori the calculation of the extreme polarization *E_m_*. The extreme polarization is the maximum value of the potential transients once the access voltage is subtracted. Figure 7 shows the original potential transient (black line) and the corrected potential transient (red line) once the contribution of the access voltage is eliminated.

To allow the user to graphically verify that the access voltage has been correctly calculated, we have developed and implemented an algorithm that graphically corrects the potential transients (Figure 7). The graphical correction implies that, once the access voltage is calculated (by using either method 1 or method 2), the result is subtracted from the potential transient, yielding the so-called “corrected potential transient”.

**Figure 8:**
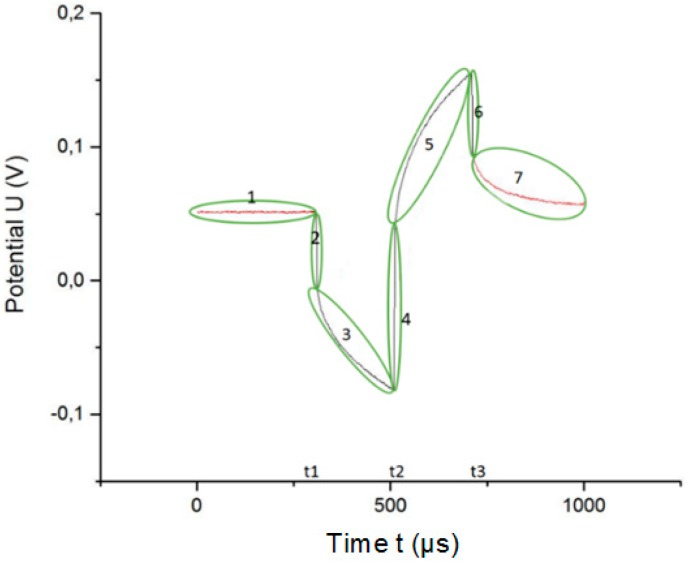
The voltage transient is divided into seven parts to graphically correct the access voltage.

To eliminate the access voltage from the potential transient, the potential transient is divided into seven parts, as shown in Figure 8. If $f\left(x_{i}\right)$ describes the potential of the WE with respect to the RE at each moment of time $x_{i}$ and $f^{\prime }\left(x_{i}\right)$ is the time derivation of $f\left(x_{i}\right)$, then each moment of time $x_{i}$ belongs to one of the seven parts, if the following conditions are fulfilled:

(11) $$x_{i}\in \{\begin{array}{cc} \mathrm{part}\;1 & \;\mathrm{if}\;f^{\prime }\left(x_{i}\right)\geq \mathrm{lower}\;\mathrm{limit}\;\mathrm{and}\;x_{i}<t_{1}\\ \mathrm{part}\;2 & \;\mathrm{if}\;f^{\prime }\left(x_{i}\right)<\mathrm{lower}\;\mathrm{limit}\;\mathrm{and}\;x_{i}<t_{2}\\ \mathrm{part}\;3 & \;\mathrm{if}\;f^{\prime }\left(x_{i}\right)\geq \mathrm{lower}\;\mathrm{limit}\;\mathrm{and}\;t_{1}<x_{i}<t_{2}\\ \mathrm{part}\;4 & \;\mathrm{if}\;f^{\prime }\left(x_{i}\right)>\mathrm{higher}\;\mathrm{limit}\;\mathrm{and}\;t_{1}<x_{i}<t_{3}\\ \mathrm{part}\;5 & \;\mathrm{if}\;f^{\prime }\left(x_{i}\right)\geq \mathrm{lower}\;\mathrm{limit}\;\mathrm{and}\;x_{i}<t_{3}\\ \mathrm{part}\;6 & \;\mathrm{if}\;f^{\prime }\left(x_{i}\right)<\mathrm{lower}\;\mathrm{limit}\;\mathrm{and}\;x_{i}>t_{2}\\ \mathrm{part}\;7 & \;\mathrm{if}\;f^{\prime }\left(x_{i}\right)\geq \mathrm{lower}\;\mathrm{limit}\;\mathrm{and}\;x_{i}>t_{3} \end{array}$$

If $g\left(x_{i}\right)$ describes the corrected potential of the WE with respect to RE at each moment of time $x_{i}$, then the corrected potential is the potential after the access voltage has been removed from the original waveform. The function $g\left(x_{i}\right)$ is:

(12) $$g\left(x_{i}\right)=\{\begin{array}{c} f\left(x_{i}\right)\;\mathrm{if}\;x_{i}\in \mathrm{part}\;1\;\mathrm{or}\;7\\ g\left(x_{i-1}\right)\;\mathrm{if}\;x_{i}\in \mathrm{part}\;2,4\;\mathrm{or}\;6\\ f\left(x_{i}\right)+V_{a1}\;\mathrm{if}\;x_{i}\in \mathrm{part}\;3\\ f\left(x_{i}\right)-V_{a3}\;\mathrm{if}\;x_{i}\in \mathrm{part}\;5 \end{array}$$

Ideally, this piecewise function describes the corrected potential. However, in practice it does not, because some points $x_{i}$ do not meet the predetermined conditions because of the noise or instability of the original waveform. However, several exceptions apply:

(1) The first point of the sample ($x_{i}=0)$ may not belong to part 1 due to the instability of the waveform and it indeed is.
(2) Some points do not meet the necessary conditions to belong to any part. After the correction, these points are equal to zero. For this reason, an intermediate function must be used to calculate the corrected potential.

Computationally, the function $g\left(x_{i}\right)$ is calculated through an intermediate function known as $h\left(x_{i}\right)$:

(13) $$h\left(x_{i}\right)=\{\begin{array}{cc} f\left(x_{i}\right) & \;\mathrm{if}\;x_{i}=0\\ f\left(x_{i}\right) & \;\mathrm{if}\;x_{i}\in \mathrm{part}\;1\;\mathrm{or}\;7\\ g\left(x_{i-1}\right) & \;\mathrm{if}\;x_{i}\in \mathrm{part}\;2,4\;\mathrm{or}\;6\\ f\left(x_{i}\right)+V_{a1} & \;\mathrm{if}\;x_{i}\in \mathrm{part}\;3\\ f\left(x_{i}\right)-V_{a3} & \;\mathrm{if}\;x_{i}\in \mathrm{part}\;5 \end{array}$$

(14) $$g\left(x_{i}\right)=\{\begin{array}{c} h\left(x_{i}\right)\;\mathrm{if}\;h\left(x_{i}\right)\neq 0\\ h\left(x_{i-1}\right)\;\mathrm{if}\;h\left(x_{i}\right)=0 \end{array}$$

By using method 1, this correction is the same as that of method 2, with the exception that the values $V_{a1}$ and $V_{a3}$ are replaced by $V_{Rs}$.

### 2.3. Calculation of the Maximum and Minimum Polarization Potentials

The VT allows determining the maximum polarization (i.e., the most negative and most positive potentials $E_{mc}$ and $E_{ma}$, respectively, across the electrode/electrolyte interface). The maximum polarization is attained when either $E_{mc}$ or $E_{ma}$ exceeds the water window. $E_{mc}$ and $E_{ma}$ are calculated by using Equation (4). $V_{\max}$ and $V_{\min}$ are the maximum and minimum values of the function $f\left(x_{i}\right)$, avoiding the first and last 15 µs of the sample: $V_{\max}=\max[f\left(x_{i}\right)$], $V_{\min}=\min\left[f\left(x_{i}\right)\right]$. The interpulse potential $E_{ipp}$ is the WE interpulse potential with respect to the RE and is calculated as the average of the points in the first interpulse period:

(15) $$E_{ipp}=\overset{x_{i}=t_{1}-ft}{\underset{x_{i}=ft}{\sum }}\frac{f\left(x_{i}\right)}{N},$$

where *N* is the number of points in the closed interval $\left[ft,t_{1}-ft\right]$.

### 2.4. Calculation of the Maximum Reversible Charge Injection Capacity

The CIC is the maximum charge injection capacity $Q_{inj}$ that can be delivered without exceeding the limits of the electrochemical potential window (*E_a_* and *E_c_*). $Q_{inj}$ is calculated for each value of the maximum polarization potential $E_{m}$. For a stimulation signal that is a symmetric square, $Q_{inj}$ is:

(16) $$Q_{inj}=i\frac{p_{w}}{A},$$

where $Q_{inj}$ is the charge injection capacity (µC/cm^2^), i is the amplitude of the stimulation current (A), $p_{w}$ is the pulse width of the stimulation signal (µs), and *A* is the geometric surface area of the electrode (cm^2^).

**Figure 9:**
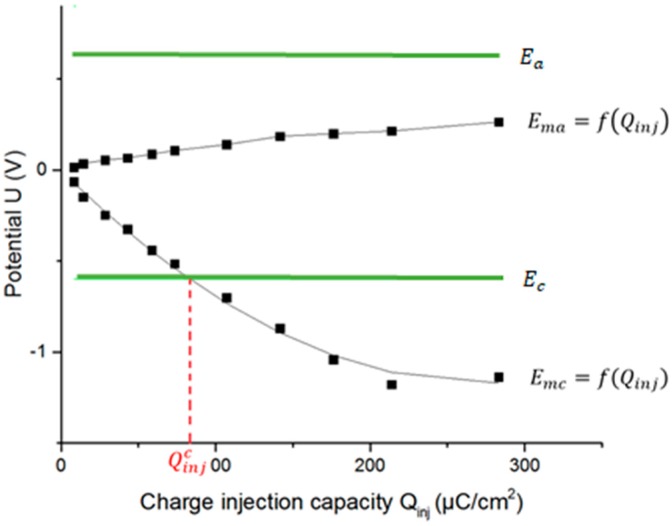
Determination of CIC for electrode A by calculating intersections of functions $E_{ma}=f\left(Q_{inj}\right)$ and $E_{mc}=f\left(Q_{inj}\right)$ with their relevant electrochemical potential window limits $(E_{a}$ and $E_{c})$.

There are two dependent variables ($E_{ma}$ and $E_{mc}$) and one independent variable $Q_{inj}$. Next, it is possible to determine the functions $E_{ma}=f\left(Q_{inj}\right)$ and $E_{mc}=f\left(Q_{inj}\right)$ and to calculate $Q_{inj}$ when $E_{ma}=E_{a}$ and $E_{mc}=E_{c}$. When this function intersects its related electrochemical potential window line, $Q_{inj}$ becomes the so-called $Q_{inj}^{*}.$ The lower positive value of the previously calculated $Q_{inj}^{*}$ is the CIC (see Figure 9).

Many electrodes are observed to behave as linear or second-order functions. For this reason, the estimates of the relationships between variables (functions $E_{ma}=f\left(Q_{inj}\right)$ and $E_{mc}=f\left(Q_{inj}\right)$) are determined by a regression analysis. However, the characteristics of some electrodes are not well described by the aforementioned regressions, so the relation is described by a piecewise linear function. The regression models and the piecewise linear function predict the value of an independent variable ($Q_{inj}$) given the known value of the dependent variable ($E_{ma}$ or $E_{mc}$), if the independent variable is within the range of values in the dataset (interpolation) or even if it is not (extrapolation).

## 3. Results and Discussion

This section describes and discusses the results of the CV and VT measurements performed with the microelectrodes listed in Table 1. Automated calculations were done by using the program that we developed on the ORIGIN platform. This program allows performing a comparative study of the CIC of an electrode immersed in an electrolyte at constant temperature based on the pulse width of the excitation signal. At the end of the analysis, a report is automatically created showing the parameters of the test, including the CSC_c_ from CV and the CIC obtained for each pulse width.

A. Results from cyclic voltammetry

**Figure 10:**
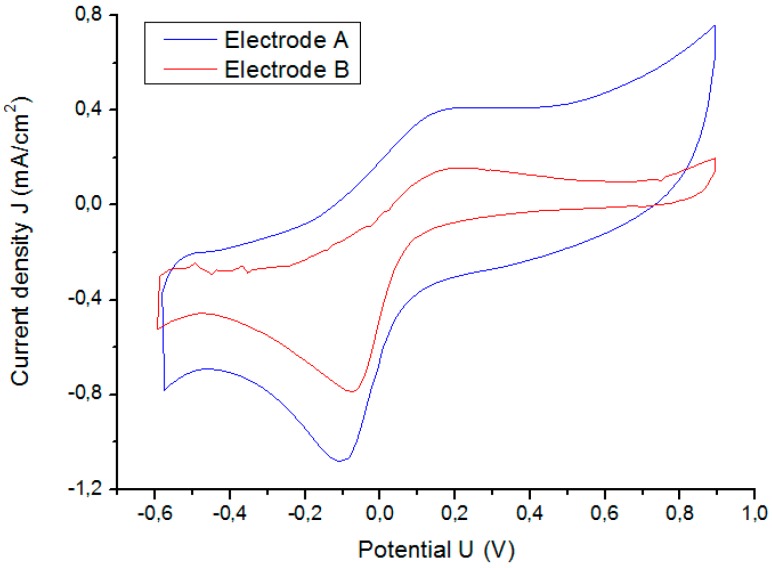
Comparison of results of cyclic voltammetry for Pt microelectrodes A and B (see Table 1). Scan rate was 100 mV/s.

CV determines the electrochemical potential window and the CSC_c_. However, although the electrochemical reactions of Pt do not change, and the rate at which the applied potential is scanned remains the same, the response of CV differs depending on the GSA of the electrode as shown in Figure 10.

Figure 10 shows that the boundaries of the electrochemical potential window for A and B in an isotonic saline solution are −0.6 and +0.9 V. Results for electrode C are reported in section C. The CSC_c_ is defined as the total available reversible charge in a stimulation pulse. A CSC_c_ of 6.26 (3.70) mC/cm^2^ is obtained for electrode A (B). The CSC_c_ depends on the rate at which the potential is swept, so its use is limited for predicting the CIC of an electrode because the CSC_c_ is calculated near equilibrium conditions.

B. Results from voltage transient measurements

The access voltage is calculated by using the conventional method (method 1) and by applying the proposed method (method 2), to compare the two methods and to determine the advantages and limitations of the proposed method. Results for electrode B as the WE are exemplary shown in this section, because the analysis of the other two electrodes A and C is similar.

### 3.1. Calculation of the Maximum Reversible Charge Injection Capacity

**Figure 11:**
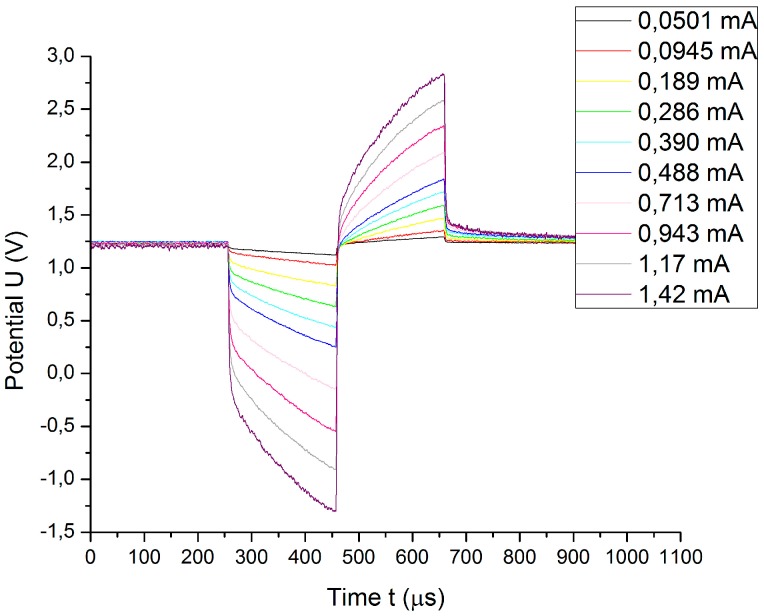
Potential transients of electrode B obtained by applying charge-balanced biphasic symmetric pulses with different current amplitudes and 200 µs pulse width.

Figure 11 shows the potential transients of electrode B obtained by the application of charge-balanced biphasic symmetric pulses with amplitudes ranging from 0.05 to 1.88 mA and a pulse width of 200 µs.

**Figure 12:**
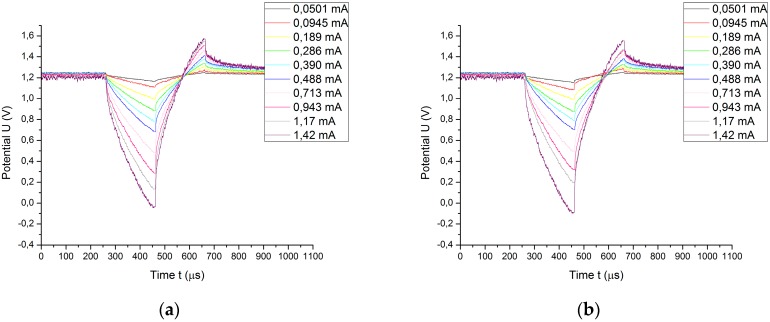
Potential transients of electrode B after the access voltage is subtracted. (**a**) Access voltage calculated by using conventional method (method 1). (**b**) Access voltage calculated by using proposed method (method 2).

Once the access voltage is calculated by using method 1 and method 2, the potential transients are graphically corrected, subtracting the pertinent access voltage. These results are depicted in Figure 12.

**Figure 13:**
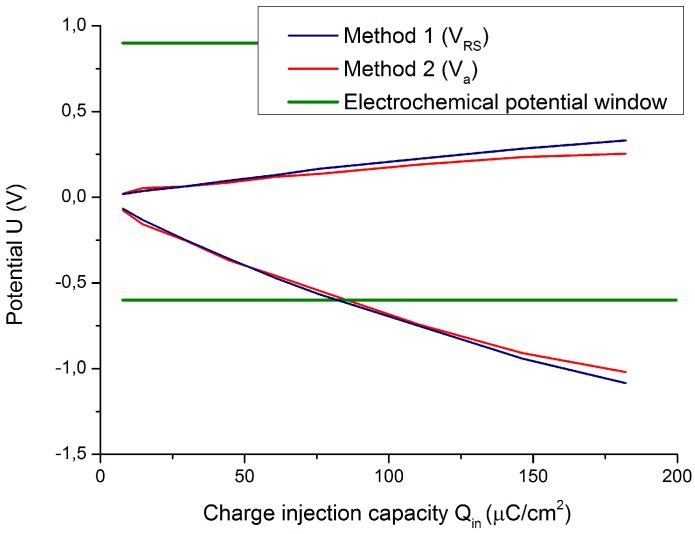
Extreme polarization values for electrode B calculated by method 1 (blue line) and method 2 (red line) as a function of injected charge.

Using either the conventional calculation method or the proposed method, no significant differences occur in the potential transients. However, the access voltages are not equal, so the extreme polarization potentials vary. The differences between both methods are shown in Figure 13, in which the extreme polarization potentials are shown as a function of the injected charge per unit area.

The extreme polarization potential calculated by method 2 is slightly less than that calculated by method 1 because method 2 does not approximate the access voltage as the voltage drop in the electrolyte, but also considers the overpotential terms. Higher access voltages imply lower polarization, so more reversible delivered charge ($CIC_{M1}$ = 80.38 µC/cm^2^ to $CIC_{M2}$ = 81.63 µC/cm^2^). Both methods are suitable and give similar results, with a relative error of 1.53%.

### 3.2. Comparison of Access Voltage Calculation

Table 2 shows the relationship between the different access voltages and the current amplitude of the excitation signal for electrode B. *V_Rs_* is the voltage drop in the electrolyte calculated by using method 1. $V_{a1}$ is the access voltage at the beginning of the pulse, and $V_{a3}$ is the access voltage at the end of the pulse (both calculated by using method 2, see Figure 6).

The voltage drop $V_{Rs}$ in the electrolyte corresponds to 95–100% of the access voltage $V_{a}$ for electrode B, while in previous studies done on microelectrodes made of conically shaped activated iridium oxide it typically corresponded to 80% of $V_{a}$ [41].

**Figure 14:**
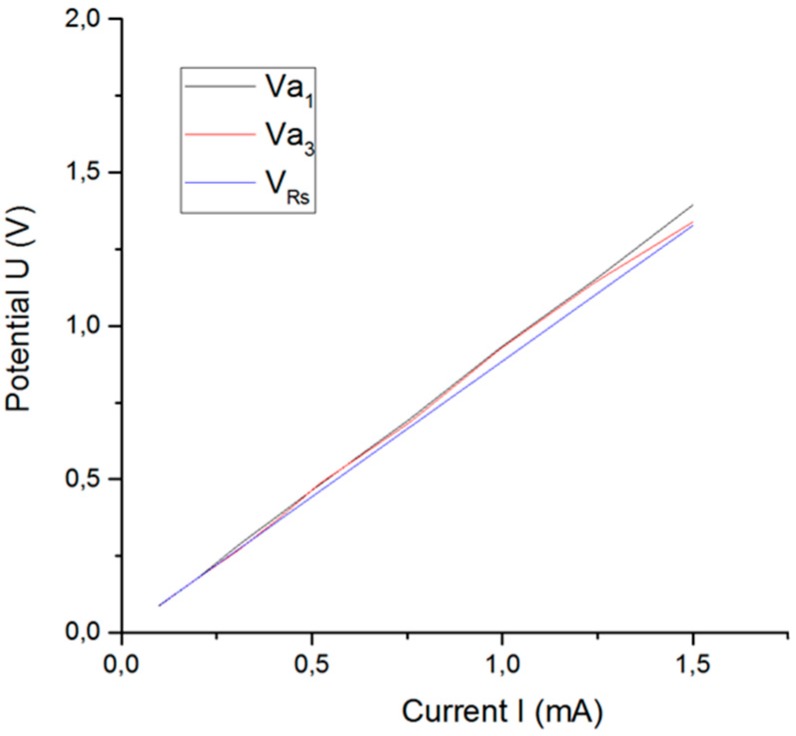
Access voltage as a function of current amplitude of excitation signal.

Figure 14 shows that the access voltage taken at the beginning of the current pulse ($V_{a1}$) differs from the access voltage taken at the end of the pulse $(V_{a3}$). The differences between the access voltages calculated at the beginning and at the end of the pulse have been studied for monophasic [15] and biphasic stimulation pulses [33]. The overpotential terms increase with increasing current amplitude, with the result that the difference between $V_{Rs}$ and $V_{a}$ also increases.

### 3.3. Reproducibility

**Figure 15:**
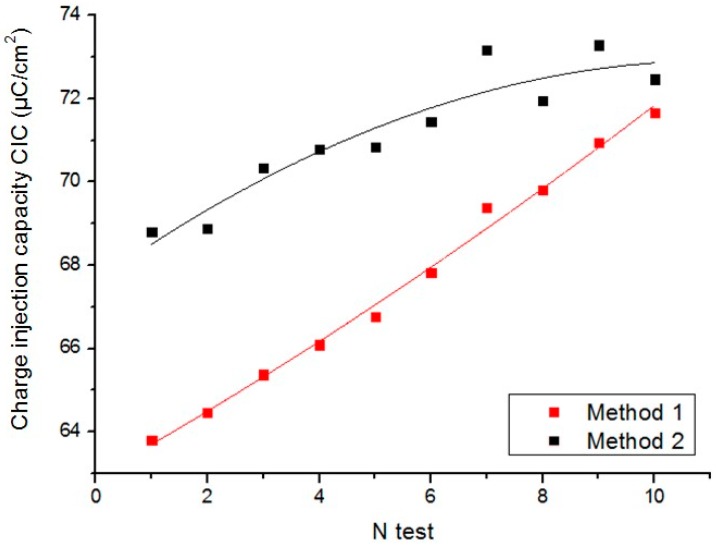
Maximum reversible charge injection capacity of electrode B obtained with *N* = 10 consecutive measurements.

The CIC of an electrode is a complex measure because it depends on several factors: material, shape, ESA and GSA of the electrode, composition and temperature of the electrolyte, and the characteristics of the excitation signal (frequency, pulse width, and waveform). A reproducibility study was made by using electrode B to examine the differences between the two proposed methods and the stability of the electrode. Figure 15 shows the graph of the results for CIC from Table 3 obtained by applying both methods for ten consecutive measurements using electrode B.

The electrode was immersed in the solution for the total duration of the experiment to keep the position of the electrodes fixed. However, the electrolyte was penetrating the electrode during the entire experiment, thus increasing the ESA of the electrode. Conversely, the CIC is very sensitive to small thermal changes, and increases with increasing temperature [15]. The temperature increases slightly (<0.1 °C) because of the flow of current from one electrode to another. Figure 15 shows that the CIC increases with increasing ESA of the electrode and electrolyte temperature. 

Method 2 is characterized by a standard deviation less than that obtained with method 1 (1.59 vs. 2.75). However, Figure 15 confirms that method 2 is more influenced by the noise of the original signal because the access voltage is calculated by analyzing the derivation. The relative error between both methods for the ten measurements shown is in the range of 1.12–7.30%.

C. Increase of charge injection capacity due to increase in roughness factor

The surface roughness of the electrodes strongly influences the CIC [42]. We compare the electrochemical properties of two electrodes with the same shape and size and made of the same material, but with different surface roughness, namely electrode B and electrode C (coated by electrodeposition with several layers of microporous Pt). 

**Figure 16:**
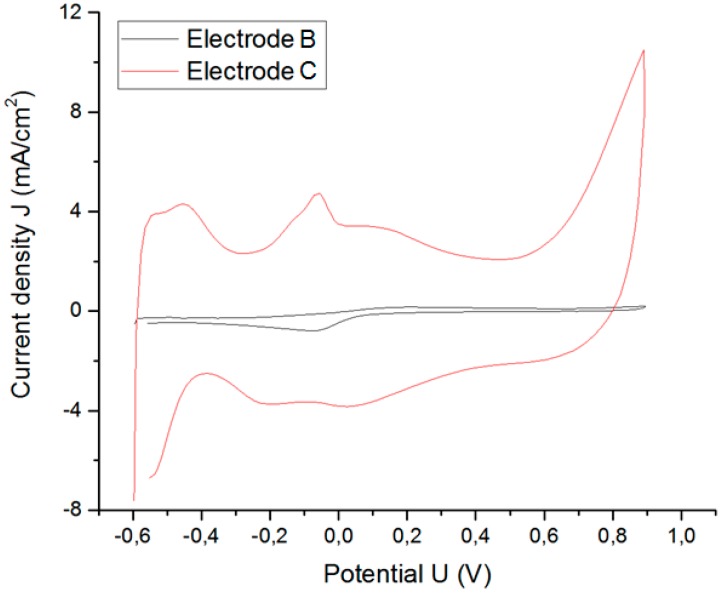
Increase in CSC_c_ due to increase in surface roughness.

Figure 16 shows the CV results for both electrodes. The area under the curve of electrode C increases considerably (cathodic current density) with respect to the uncoated electrode B. The CSC_c_ increases from 3.7 to 37.7 mC/cm^2^ due to the microporous coating of electrode C. 

**Figure 17:**
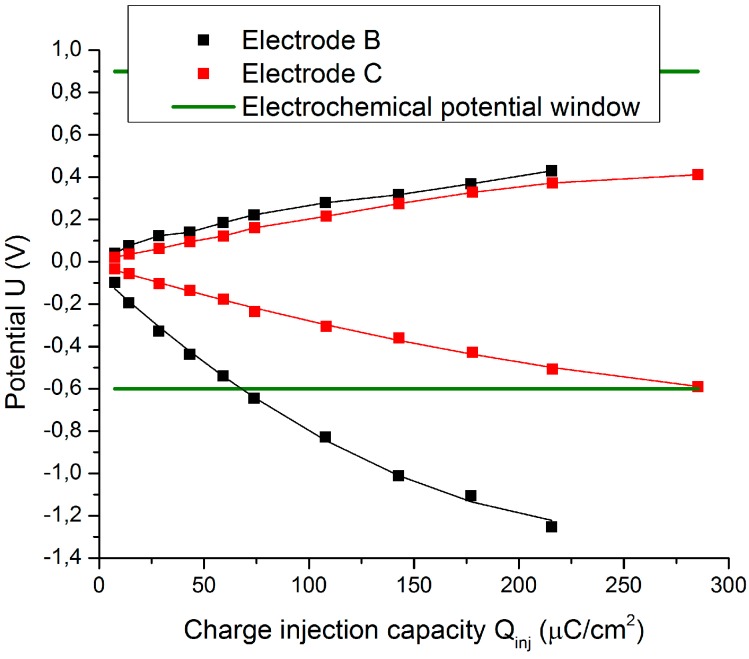
Increase in CIC due to higher surface roughness for a pulse width of 200 µs.

Figure 17 shows the extreme polarization potentials obtained by method 2 for electrodes B and C. The extreme polarization potentials for the same injected charge are lower for the microporous electrode C, which leads to an increase in the CIC. The CIC is 81.63 µC/cm^2^ for the uncoated electrode B, whereas it is 295.90 µC/cm^2^ for the microporous electrode C.

D. Comparison with other studies

This section compares the results obtained by applying the proposed method 2 with the results found in the literature. Table 4 shows the CIC calculated by method 2 and the CSC_c_ of electrodes A, B, and C. The CIC is calculated from a VT measurement made with a 200 µs pulse width, while the CSC_c_ is obtained from CV with a scan rate of 100 mV/s. The highest CIC was measured for the coated electrode C. A large reversibly delivered charge available also occurs for the sputtered Pt electrode B. The lowest reversibly delivered charge available occurs for the sputtered Pt electrode A with larger dimensions.

Rose and Robblee [35] determined the Pt charge-injection limits for stimulation with cathodic-first 0.2 ms pulses to be 50–150 µC/cm^2^ (GSA = 9.5 × 10^−3^ to 1.3 × 10^−4^ cm^2^). Poppendieck et al. [34] performed VT measurements with the same stimulation parameters as Rose and Robblee and determined CIC = 64 µC/cm^2^ and 524 µC/cm^2^ for Pt and microporous Pt electrodes (radius = 150 µm), respectively. The CIC for Pt macroelectrodes (GSA = 2 × 10^−3^ to 2.3 × 10^−3^ cm^2^) is reported to be 34–54 µC/cm^2^ with a pulse width of 100–3200 µs [33].

The literature values for CSC_c_ for Pt electrodes vary from 2.92 to 26.6 mC/cm^2^ [43]. Cuff Pt electrodes were characterized as having CSC_c_ = 4 mC/cm^2^ and CIC = 75 µC/cm^2^ (GSA = 1 mm^2^) [22].

The increase in the CSC_c_ with increasing surface roughness has been studied previously [44]. Bare Pt electrodes with a diameter of 1.83 mm were characterized as having a CSC_c_ = 0.9 mC/cm^2^. After being coated with nanowires, the electrodes reached 1.34 mC/cm^2^. Pt electrodes with a diameter of 250 μm were characterized as having a CSC_c_ = 2.1 mC/cm^2^, and these same electrodes with surface roughness greater than unity had a CSC_c_ = 10.4 mC/cm^2^ [36].

The range of published values for CSC_c_ and CIC summarized in Table 5 for Pt electrodes is consistent with the values obtained in this study.

## 4. Conclusions

In this paper, we describe the development of a measurement setup and an automated calculation method to determine the CIC of implantable microelectrodes. Knowledge of the CIC is vital to design safe stimulation protocols: it provides information on the effectiveness of the stimulation and allows determining the maximum current amplitude of the stimulation signal that can be applied to the electrode without damaging the electrode or the target tissue.

We proposed and implemented a method to calculate the access voltage that overcomes the limitations of conventional methods. The proposed method provides the following improvements with respect to the conventional methods:

(i) The access voltage can be calculated without previous knowledge of the electrolyte (or excitable tissue) resistance, which constitutes an important improvement for in vivo experimentation, where tissue resistivity is not known with precision and varies after electrode implantation.
(ii) It can be applied to large, porous, or coated electrodes, for which Equation (6) for calculating the electrolyte resistance is not valid.
(iii) It does not neglect the overpotential terms, which gives a more precise result for the access voltage.
(iv) No interpulse period needs to be introduced between the cathodic and anodic phase of the biphasic pulse.

To compare and validate the results obtained with the proposed method, we implemented a conventional method for calculating the CIC. The results obtained using both methods are consistent with each other. In addition, the CIC is slightly greater for the proposed method because it includes the overpotential terms, which leads to greater precision in the calculation of the access voltage because it accounts for the fact that the access voltage depends on the pulse polarization applied to the electrode. However, the proposed method is more sensitive to external noise, because noise from potential transients is amplified by taking the derivation in the calculation of the access voltage.

## Figures and Tables

**Table 1:** Pt microelectrodes characterized in present study.

Microelectrode	Type	Material	GSA (cm^2^)
A	Planar	Sputtered Pt	0.001963
B	Cuff	Sputtered Pt	0.0013
C	Cuff	Microporous Pt	0.0013

**Table 2:** Relationship between access voltage and current amplitude of excitation signal for electrode B.

Current (mA)	*V_Rs_*/*V_a_*_1_	*V_Rs_*/*V_a_*_3_
0.09955	1.00	0.99
0.19942	1.00	1.00
0.30267	0.95	1.00
0.41316	0.95	0.98
0.51667	0.95	0.95
0.75474	0.96	0.98
0.99733	0.95	0.95
1.24186	0.96	0.96
1.50906	0.95	0.99

**Table 3:** Maximum reversible charge injection capacity (CIC in µC/cm^2^) for electrode B calculated from ten consecutive measurements.

Test	1	2	3	4	5	6	7	8	9	10
**Method 1: *CIC_M_*_1_**	63.79	64.46	65.37	66.09	66.77	67.83	69.38	69.8	70.94	71.66
**Method 2: *CIC_M_*_2_**	68.81	68.87	70.33	70.79	70.83	71.45	73.16	71.96	73.29	72.47

**Table 4:** Results for the three electrodes of this study.

Electrode	Type	Material	GSA (cm^2^)	CIC_M2_ (µC/cm^2^)	CSC_c_ (mC/cm^2^)
A	Planar	Sputtered Pt	0.001963	26.06	6.26
B	Cuff	Sputtered Pt	0.0013	81.63	3.70
C	Cuff	Microporous Pt	0.0013	295.90	37.67

**Table 5:** Results from other studies.

Reference	Material	GSA (cm^2^)	CIC (µC/cm^2^)	CSC_c_ (mC/cm^2^)
[35]	Sputtered Pt	0.0095–0.00013	50–150	-
[34]	Sputtered Pt	0.0007	64	-
[34]	Microporous Pt	0.0007	524	-
[33]	Sputtered Pt	0.002–0.0023	34–54	-
[43]	Sputtered Pt	-		2.92–26.6
[22]	Sputtered Pt	0.0010	75	4
[45]	Bare Pt	0.1052	-	0.9
[45]	Coated Pt	0.1052	-	1.34
[45]	Sputtered Pt	0.0019	-	2.1
[45]	Coated Pt	0.0019	-	10.4
